# Investigation on antigangliosides antibodies in asymptomatic HIV patients

**DOI:** 10.1186/1471-2334-14-S4-P25

**Published:** 2014-05-29

**Authors:** Ilinca Nicolae, Corina Daniela Ene Nicolae, Emanoil Ceauşu

**Affiliations:** 1 Clinical Hospital of Infectious and Tropical Diseases “Dr. Victor Babeş”, Bucharest, Romania; 2 Carol Davila University of Medicine and Pharmacy, Bucharest, Romania

## 

Antigangliosides antibodies were observed in neoplastic diseases, bacterial and viral infections, autoimmune diseases and neurological disorders. Objective: assessment of antigangliosides antibodies anti -GM1, -GM2, -GM3, -GD1a, -GD1b, -GT1b, -GQ1b of IgG type in asymptomatic HIV patients.

The study was based on the prospective analysis of 32 patients with asymptomatic HIV infection, with no retroviral treatment, without altered neurological status associated with the disease and 48 healthy subjects.

Antigangliosides antibodies were determined by immunoblot method, using Euroline kits.

In healthy subjects, antigangliosides antibodies of IgG type against all the mentioned gangliosides were negative.

In patients with HIV infection, antigangliosides antibodies of IgG type had the following frequency: 6.2% anti-GM1, 15.7% anti-GM2, 12.5% anti-GM3, 18.7% anti-GD1a, 6.2% anti-GD1b, 9.4% anti-GT1b, 18.7% anti-GQ1b.

The statistical analysis showed a significant difference between anti-GM2, anti-GD1a and anti-GQ1b status in HIV group compared with the control group.

The authors considered that gangliosides expressed on the membrane of HIV infected cells induced antigangliosides antibodies’ synthesis. Antigangliosides antibodies’ presence seems to be a primary immunological event in HIV infection and might play a physiopathological role in the studied viral infection.

